# Correction: Realization of multi-configurable logic gate behaviour on fluorescence switching signalling of naphthalene diimide congeners

**DOI:** 10.1039/d1ra90169a

**Published:** 2021-11-17

**Authors:** Hridoy Jyoti Bora, Pranjal Barman, Sushanta Bordoloi, Gautomi Gogoi, Bedanta Gogoi, Neelotpal Sen Sarma, Anamika Kalita

**Affiliations:** Physical Sciences Division, Institute of Advanced Study in Science and Technology Paschim Boragaon Guwahati-781035 Assam India anamik.kalita01@gmail.com; Department of Electronics and Communication Technology, Gauhati University Guwahati-781014 Assam India; Department of Electronics and Communication Engineering, National Institute of Technology Mizoram Aizawl-796012 India; Department of Chemistry, Gauhati University Guwahati-781014 Assam India

## Abstract

Correction for ‘Realization of multi-configurable logic gate behaviour on fluorescence switching signalling of naphthalene diimide congeners’ by Hridoy Jyoti Bora *et al.*, *RSC Adv.*, 2021, **11**, 35274–35279. DOI: 10.1039/d1ra06728a.

The authors regret that there was an error in the sentence in lines 24–27 in the left column on page 35278 of the original article. The text originally read, “Mass spectrometric data Shimadzu UV-Vis spectrophotometer, UV-2600 is used to record the absorption spectra were gained from a THERMO 3000 Exactive Plus Orbitrap Mass Spectrometer”. This sentence should read, “Mass spectrometric data were gained from a THERMO 3000 Exactive Plus Orbitrap Mass Spectrometer. Shimadzu UV-Vis spectrophotometer, UV-2600 is used to record the absorption spectra”.

The Royal Society of Chemistry apologises for these errors and any consequent inconvenience to authors and readers.

## Supplementary Material

